# Five-Year Outcomes of Deep Sclerectomy in Pseudoexfoliation Glaucoma Compared to Primary Open-Angle Glaucoma

**DOI:** 10.3390/jcm13237434

**Published:** 2024-12-06

**Authors:** Carlo Fiore, Xiao Shang, Joel-Benjamin Lincke, Nathanael Urs Häner, Martin Sebastian Zinkernagel, Jan Darius Unterlauft

**Affiliations:** 1 Department of Ophthalmology, Inselspital, Bern University Hospital, University of Bern, Freiburgstrasse 18, 3010 Bern, Switzerland; 2 Graduate School for Cellular and Biomedical Sciences, University of Bern, Mittelstrasse 43, 3012 Bern, Switzerland

**Keywords:** glaucoma, deep sclerectomy, primary open-angle glaucoma, pseudoexfoliation glaucoma, glaucoma surgery outcomes, retinal nerve fiber layer

## Abstract

**Objectives**: This study aimed to investigate the five-year outcomes of deep sclerectomy (DS) in patients with pseudoexfoliation glaucoma (PEXG) and primary open-angle glaucoma (POAG). **Methods**: This retrospective, observational, unicentric study analyzed POAG and PEXG patients. Intraocular pressure (IOP), the number of IOP-lowering medications, peripapillary retinal nerve fiber layer (RNFL) thickness, the number of postoperative interventions, surgical success rates, and secondary surgery rates were evaluated at baseline and during follow-up appointments. **Results**: A total of 109 POAG and 153 PEXG eyes were included. Over the 5-year follow-up, IOP decreased in both groups (*p* = 0.17), from 22.8 ± 0.7 to 13.3 ± 0.6 mmHg (*p* < 0.001; POAG) and from 24.3 ± 0.8 to 16.6 ± 1.2 mmHg (*p* < 0.001; PEXG). The number of IOP-lowering medications decreased comparably (*p* = 0.99), from 3.1 ± 0.1 to 1.7 ± 0.3 (*p* = 0.001; POAG) and from 3.4 ± 0.1 to 1.7 ± 0.2 (*p* < 0.001; PEXG). Peripapillary RNFL thickness decreased in both groups (*p* = 0.31), from 60.6 ± 1.9 to 54.2 ± 2.4 µm (*p* < 0.001; POAG) and from 63.1 ± 1.7 to 58.0 ± 2.3 µm (*p* < 0.001; PEXG). The 5-year complete success rates were 33% and 12% for the POAG and PEXG groups, respectively (*p* = 0.01). The qualified success rates were 63% and 40% (*p* = 0.03). Secondary glaucoma surgery was required in 8% of POAG eyes and 21% of PEXG eyes (*p* = 0.04). **Conclusions**: DS resulted in comparable results for IOP, medications, and RNFL development in the PEXG and POAG groups but in less favorable outcomes concerning surgical success and further necessary repeated glaucoma surgery in patients with PEXG over the 5-year follow-up period.

## 1. Introduction

Glaucoma is a cluster of medical conditions with diverse pathophysiological processes, sharing progressive optic disc cupping with retinal ganglion cell loss and consequent visual field defects [1]. These changes are strongly related but apparently not exclusively due to intraocular pressure (IOP) [2]. Primary open-angle glaucoma (POAG) is characterized by an open anterior chamber angle without any secondary disease etiology and is the most frequent type of glaucoma in Europe and Northern America, with a prevalence in people aged 40–80 years of 2.51% and 3.29%, respectively [3,4]. Pseudoexfoliation glaucoma (PEXG) is due to pseudoexfoliation syndrome (PEX), a systemic disorder that leads to abnormal deposits composed predominantly of fibrillary material [5]. The prevalence of PEX has been reported to vary widely between regions, ranging from 4% to 21% in different European countries [6]. These deposits can result in the degeneration of Schlemm’s canal and aqueous humor outflow obstruction in the trabecular meshwork, increasing IOP and inducing glaucoma [7]. A number of topical and systemic medications, as well as laser and surgical procedures, can help reduce IOP [8], which as of today, is the only proven effective treatment to decelerate or, at best, stop disease progression [2]. Usually, conservative approaches are preferred initially. Though multiple classes of medications exist, failure to meet IOP targets and disease progression despite maximally tolerated conservative treatment can indicate the need for interventional surgical therapy [8].

One of the first choices is trabeculectomy (TE) [9], a technique which functions through the creation of a direct transscleral fistula for the aqueous humor to reach the subconjunctival space [10]. This approach has been observed to be prone to complications [11], leading to the search for less invasive and safer techniques leading to similar results. Deep sclerectomy (DS) is a surgical procedure developed with this intent. It increases aqueous humor outflow from the anterior chamber into the subconjunctival space under a scleral flap [12]. Its aim is to provide substantial IOP reduction with fewer complications than TE [13].

PEXG is associated with faster progression compared to POAG [14]. The amount of deposited pseudoexfoliation material in the aqueous humor outflow system has been observed to be related to disease severity [15]. Considering this and the pathomechanism behind PEXG, non-penetrating filtering surgery such as DS may not be the perfect option in the long term, since the trabecular meshwork is left in place and congestion by pseudoexfoliation material is proceeding. As of today, DS has only been observed to be effective for PEXG patients in trials that either had smaller groups, shorter observation times, or both compared to our herein presented study [16,17,18,19,20,21,22].

The aim of this study was to investigate the five-year outcomes of DS for PEXG compared to POAG cases. This information could help provide targeted surgical care for these patient populations. Therefore, we retrospectively investigated the course of functional and anatomical parameters, as well as the development of complete and qualified success rates, in a comparative group of POAG and PEXG cases followed-up over the first 5 years after surgery.

## 2. Materials and Methods

This retrospective, observational, unicentric study is a sub-analysis of previously collected data from the Bern Glaucoma Registry Study (BGRS). The BGRS is a retrospective medical records review study conducted at the Department of Ophthalmology, Inselspital, Bern University Hospital. Consecutive medical records of patients over 18 years old who underwent glaucoma surgery between 2012 and 2023 were reviewed for eligibility. The BGRS was approved by the Ethics Committee of the Canton of Bern (BASEC-ID: 2022-01046) and adhered to the tenets of the Declaration of Helsinki. Informed consent for participants was waived due to the retrospective nature of this study.

Patients diagnosed with POAG or PEXG who underwent DS between July 2012 and November 2023 were included. The minimum sample size required to detect a 20% difference in surgical success rates (expected to lie at 60% and 40% for complete and qualified success for each group, respectively) with a 95% confidence interval and power of 0.80 was calculated to be 107 eyes for each cohort. Indications for glaucoma surgery were disease progression, above-target IOP on maximum tolerable medical therapy, or the inability to escalate medical therapy further due to existing allergies or other medical conditions. Disease progression was defined as a worsening of the visual field mean defect (MD) on three consecutive tests with an increase of at least −2.00 dB within one year. The decision to perform DS and not any of the other available surgical options was usually made at the discretion of the treating physician during outpatient examination. Medical records were reviewed, and clinical data were added to the BGRS database and subsequently analyzed. Eyes/patients with co-existing ocular or central nervous system diseases that could affect the ophthalmic tests performed (i.e., excessive myopia, macular degeneration, multiple sclerosis, etc.) were excluded. If both eyes of a single patient were eligible, only the first eye undergoing surgery was included to reduce the effect of data compounding due to bilateral eyes [23].

### 2.1. Surgical Procedures

Surgeries were performed by 4 experienced ophthalmic surgeons following standard surgical procedures described in previous studies [24,25]. The minimum and maximum number of years of experience with which a surgeon operated on the patients in the study was 7 and 20 years, respectively. These were defined as the time between their first year as an attending surgeon and the first or last year in which they operated on the patients included in the study. In summary, a fornix-based conjunctival flap was created. Mitomycin C (0.2 mg/mL) was applied for 2 min and meticulously rinsed thereafter with balanced salt solution. A 5 × 5 mm scleral flap was dissected, and another 2 × 2 mm deeper flap was excised to gain access to Schlemm’s canal and expose the trabeculo-Descemet window. The scleral flap was then re-approximated with two non-absorbable 10/0 interrupted sutures that were adapted according to resulting IOP. An ocular viscoelastic device (OVD) was injected beneath the scleral flap to prevent its collapse onto the scleral bed. Tenon’s capsule and conjunctiva were re-approximated to the limbus with two to four absorbable interrupted sutures. Non-absorbable mattress sutures were used to guarantee water tightness and left in situ for three weeks postoperatively. The postoperative medical plan consisted of topical tobramycin four times a day for three weeks and atropine at 0.5% twice a day for one week or equivalents. Topical prednisolone acetate or equivalent was administered four times a day for three weeks and subsequently tapered according to clinical evaluation. The follow-up plan usually included weekly examinations during the first postoperative month, with subsequent visits at 3 and 6 months and annually until 5 years postoperatively. Indications for corrective procedures were determined by the treating physician after an assessment of clinical presentation, disease course, and the resulting IOP.

### 2.2. Follow-Up Treatments and Data Collection

Data were collected from the hospital’s electronic medical records in the documentation system FIDUS (version 21.42.2, Arztservice Wente GmbH, Darmstadt, Germany). Outcomes for each case were assessed preoperatively and at the following postoperative timepoints: 1 day, 1 month, 3 months, 6 months, 1 year, 2 years, 3 years, and 5 years. Not every case was examined at each timepoint. Collected data included the diagnosis of either POAG or PEXG, patient age at surgery, sex, laterality of the treated eye, history of previous glaucoma surgeries, IOP measurements, the number of IOP-lowering medications, best corrected visual acuity (BCVA), visual field MD, peripapillary retinal nerve fiber layer (RNFL) thickness, postoperative interventions, and secondary major glaucoma surgeries after DS. IOP was measured using Goldmann applanation tonometry (Haag-Streit, Köniz, Switzerland). Both topical and systemic IOP-lowering medications were documented. For combination drugs, each active ingredient was documented as a separate medication. BCVA was measured with Snellen charts and values were converted into logMAR units. The MD of visual field tests were assessed with standard automated perimetry (SAP) using Octopus 900 (Haag-Streit, Köniz, Switzerland). Peripapillary RNFL thickness was evaluated by spectral domain optical coherence tomography (OCT) using the Spectralis OCT machine (Heidelberg Engineering, Heidelberg, Germany). Postoperative interventions included iridoplasties, bleb needlings, Nd:YAG-laser goniopunctures, 5-fluorouracil (5-FU) injections (0.1 mL subconjunctival, 50 mg/mL), and open surgical revisions. Secondary major glaucoma surgeries were defined as subsequent interventions performed to alter glaucoma progression or symptoms. These were not directly related to the management of consequences resulting from the original DS and included the following procedures: cyclophotocoagulation, evisceration, TE, insertion of a Baerveldt Glaucoma Implant, PreserFlo MicroShunt, and XEN Gel Stent. Cataract surgeries were not included in this category of procedures. Subsequent interventions, including secondary major glaucoma surgeries, were performed after thorough examination by a specialized glaucoma surgeon, deciding on the necessity for an additional surgical intervention. Resulting IOP, the number of given IOP-lowering medication hinting towards surgical failure of DS, and further functional worsening in SAP were taken into account, leading to the decision for additional glaucoma surgery (usually trabeculectomy or cyclophotocoagulation).

Surgical success was evaluated at 1, 3, and 5 years after DS surgery. Qualified success had to meet each of the following three criteria: IOP reduction equal to or greater than 20% from preoperative values, a resulting IOP lower than 21 mmHg, and no secondary major glaucoma surgery performed from the time of the DS until the evaluated timepoint. Complete success had to meet each of the three aforementioned criteria without the application of additional IOP-lowering medication.

### 2.3. Data Analysis

Clinical data were gathered and plotted using the spreadsheet applications Microsoft Excel (Version 16.83, Microsoft Corporation, Redmond, WA, USA) and Numbers (Version 13.0, Apple Inc., Cupertino, CA, USA). The software IBM SPSS Statistics (Version 29.0.2.0, IBM, Armonk, NY, USA) was used to perform the statistical analysis and to plot clinical data. Continuous variables were expressed as mean values and standard error of the mean. Categorical variables were expressed as percentages. The data were collected and analyzed by one of the operating surgeons (J.D.U.) together with a medical student (C.F.).

The primary outcomes were changes in IOP, the number of IOP-lowering medications, the number of postoperative interventions, complete and qualified surgical success rates, and the number of secondary glaucoma surgeries over the course of 5 years after surgery. Secondary outcomes were BCVA, the MD, and peripapillary RNFL thickness at each timepoint. The following statistical tests were conducted: the chi-square test to compare categorical variables, the Wilcoxon signed-rank test to compare continuous non-parametric paired variables between different timepoints in the same group, the Mann–Whitney U test to compare continuous non-parametric unpaired variables at same timepoints between groups, and the two-tailed test for the Pearson and point biserial correlation coefficients. A *p* value less than 0.05 was considered statistically significant and is shown bolded in the tables.

## 3. Results

A total of 262 eyes (109 POAG eyes, 153 PEXG eyes) that underwent DS were included in the final analysis. Baseline demographic and clinical characteristics are shown in Table 1. The POAG group was younger than the PEXG group in this study (68.8 ± 1.0 years vs. 74.2 ± 0.6 years, *p* < 0.001). There was no statistically significant difference in laterality (*p* = 0.23) and the percentage of eyes with previous glaucoma surgery (*p* = 0.67). Differences in the mean IOP (mmHg), the number of IOP-lowering medications, BCVA (logMAR), visual field MD (dB), and RNFL thickness (µm) were not statistically significant between the POAG and PEXG groups. In the POAG cohort, two TEs and one DS were performed before the DS was analyzed. In contrast, three TEs were performed prior to DS in the PEXG cohort. The percentage of males was 56% in the POAG group and 36% in the PEXG group, and a statistically significant difference was present (*p* = 0.001).

### 3.1. Intraocular Pressure

**Figure 1:**
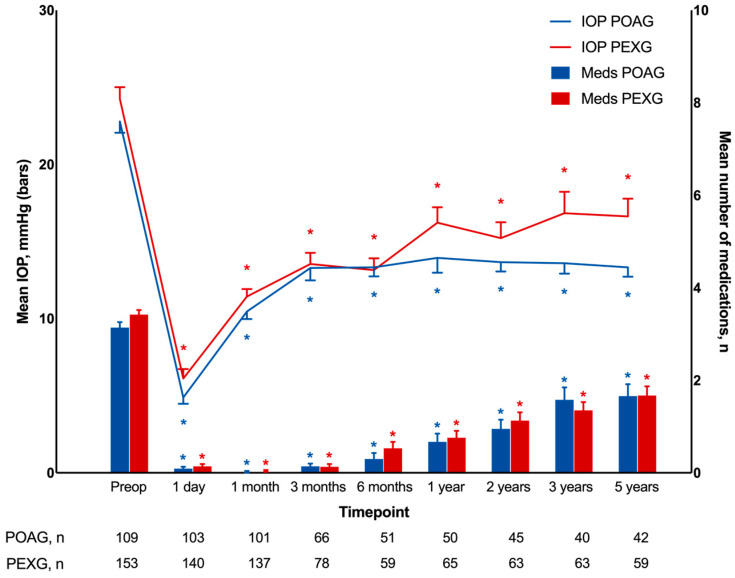
Development of mean intraocular pressure and number of glaucoma medications during 5 years of follow-up after deep sclerectomy in primary open-angle glaucoma and pseudoexfoliation glaucoma groups. IOP, intraocular pressure; POAG, primary open-angle glaucoma; PEXG, pseudoexfoliation glaucoma; Meds, number of glaucoma medications; Preop, preoperative. * indicates *p* ≤ 0.001 (Wilcoxon signed-rank test compared to preoperative values within groups).

At 1 day after surgery, the mean IOP decreased to 4.9 ± 0.4 mmHg in the POAG group and to 6.1 ± 0.6 mmHg in the PEXG group (Table 2; Figure 1). In the POAG cohort, mean IOP values increased until 3 months, while in the PEXG group, they increased until 1 year after DS surgery. Using the Wilcoxon signed-rank test, no significant differences in the mean IOP were found in the POAG cohort between 3 months and 5 years postoperatively (*p* = 0.50) and between 1 and 5 years postoperatively in the PEXG cohort (*p* = 0.50). Mean IOP values at 5 years reached 13.3 ± 0.6 mmHg and 16.6 ± 1.2 mmHg in the POAG and PEXG groups. Within groups, every difference in follow-up measurements compared to baseline was of statistical significance (*p* < 0.001), but no statistically significant differences between the POAG and PEXG cohorts were assessed at any of the analyzed timepoints. Five years after surgery, the mean IOP decrease was 41% ± 4% in the POAG group and 31% ± 6% in the PEXG group compared to baseline.

### 3.2. Number of Intraocular Pressure-Lowering Medications

At 1 day after DS, the mean number of applied IOP-lowering medications decreased to 0.1 ± 0.1 in the POAG group and to 0.1 ± 0.1 in the PEXG group (Table 2; Figure 1). Afterwards, increases in the mean number of applied IOP-lowering medications were found in both treatment groups. At 5 years after surgery, it reached 1.7 ± 0.3 and 1.7 ± 0.2 mmHg in the POAG and PEXG cohorts, respectively. Results for each follow-up visit compared to baseline values showed differences in statistical significance (*p* ≤ 0.001), but no significant difference was found between cohorts for any of the analyzed timepoints. After 5 years, the mean number of IOP-lowering medications applied was reduced by 47% ± 9% in the POAG group and 51% ± 7% in the PEXG group compared to baseline before DS.

### 3.3. Postoperative Interventions

The number of postoperative interventions in the POAG and PEXG cohorts during 5 years of follow-up after DS is shown in Table 3. As was the case for each individual procedure, no statistically significant difference between groups was observed for the total number of interventions (*p* = 0.35). Some eyes underwent a single type of corrective procedure more than once. A total of 51% of POAG eyes underwent at least one of these procedures compared to 61% of the PEXG eyes, with no significant difference between the two cohorts (chi-square test: *p* = 0.11).

### 3.4. Surgical Success Rates

**Figure 2:**
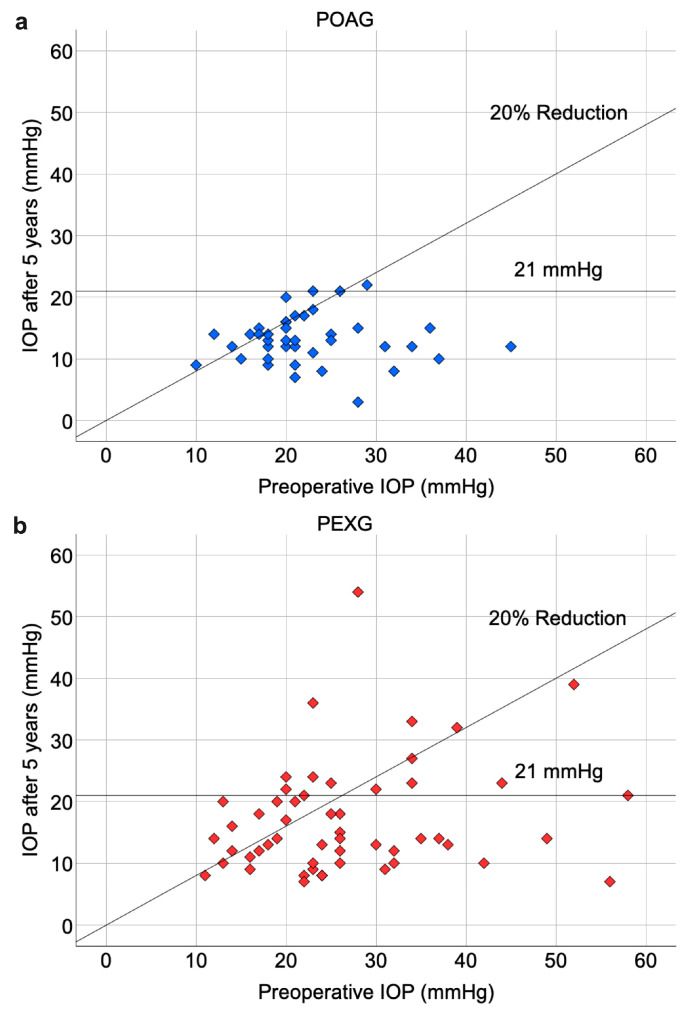
Scatter plots of IOP (mmHg) preoperatively and 5 years after deep sclerectomy in primary open-angle glaucoma (**a**) and pseudoexfoliation glaucoma (**b**) groups, including patients who underwent secondary glaucoma surgeries. Cases that achieved an IOP reduction equal to or greater than 20% from baseline values and cases that had a resulting IOP lower than 21 mmHg after 5 years are visualized under the respective lines. IOP, intraocular pressure; POAG, primary open-angle glaucoma; PEXG, pseudoexfoliation glaucoma.

The percentage of eyes with complete surgical success at 1 year was 54% for POAG and 39% for PEXG, with no significant difference (*p* = 0.11). At 3 years, success rates dropped to 29% for POAG and 28% for PEXG, which is still not significantly different (*p* = 0.89). By 5 years, 33% of POAG eyes and 12% of PEXG eyes achieved complete surgical success, with a significant difference (*p* = 0.01). At 1 year, 72% of POAG eyes and 55% of PEXG eyes achieved qualified surgical success, with no significant difference between the groups (*p* = 0.06). At 3 years, the success rates were 55% for POAG and 59% for PEXG, also not significantly different (*p* = 0.69). By 5 years, 63% of POAG eyes and 40% of PEXG eyes had qualified success, showing a statistically significant difference (*p* = 0.03). IOP changes comparing baseline to follow-up results measured 5 years after DS are visualized in two scatter plots in Figure 2.

### 3.5. Secondary Glaucoma Surgeries

In the POAG group, three cyclophotocoagulations, five TEs, one PreserFlo MicroShunt implantation, and two Baerveldt Glaucoma valve implantations were performed during the 5 years following DS. Meanwhile, nine cyclophotocoagulations, nineteen TEs, four PreserFlo MicroShunt implantations, one Baerveldt Glaucoma valve implantation, and seven XEN Gel Stent implantations were observed in the PEXG group after DS during the 5 years of follow-up. Some eyes underwent more than one of these procedures during the 5 years after DS. These cumulated to a total number of 12 secondary glaucoma surgeries in the POAG cohort and 40 in the PEXG cohort during the 5 years of follow-up, with a statistically significant difference between groups (*p* = 0.04). A total of 8% of POAG eyes underwent at least one of these procedures compared to 21% of the PEXG eyes, and a significant difference between the two cohorts was found (chi-square test: *p* = 0.01).

### 3.6. Best Corrected Visual Acuity and Visual Field Mean Defect

**Figure 3:**
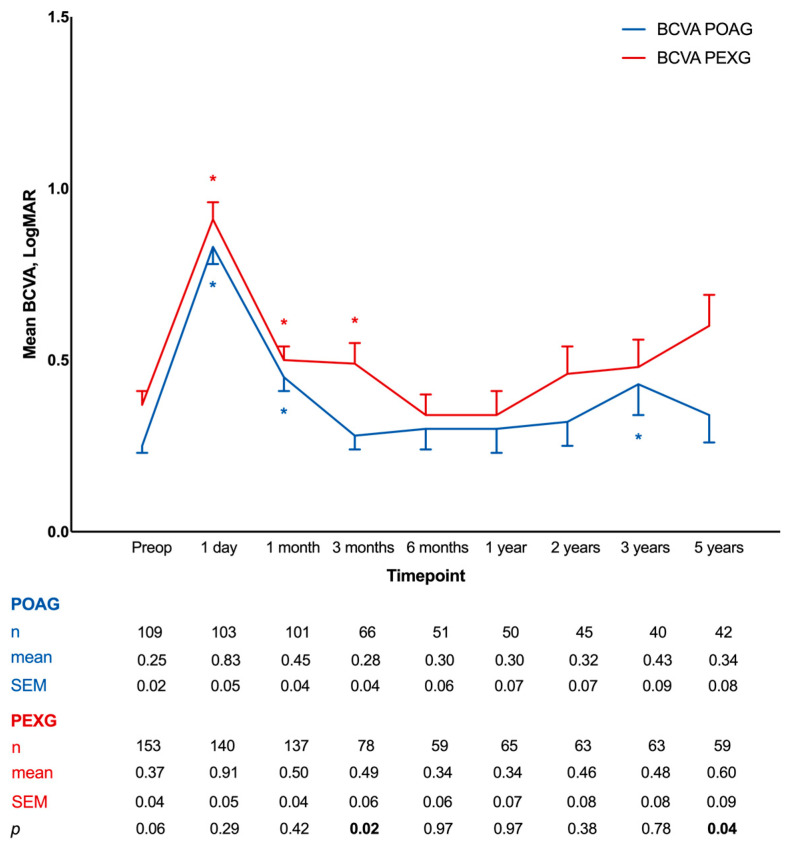
Development of best corrected visual acuity during 5 years of follow-up after deep sclerectomy in primary open-angle glaucoma and pseudoexfoliation glaucoma groups. BCVA, best corrected visual acuity; POAG, primary open-angle glaucoma; PEXG, pseudoexfoliation glaucoma; Preop, preoperative; SEM, standard mean error. Comparison between baseline and follow-ups within groups was performed using Wilcoxon signed-rank test (* indicates *p* < 0.05). Comparison between groups was performed using Mann–Whitney U test (*p* values of less than 0.05 are in bold).

The mean BCVA was 0.25 ± 0.02 logMAR and 0.37 ± 0.04 logMAR in the POAG and PEXG cohorts at baseline before surgery (Figure 3). At 1 day, the mean BCVA worsened to 0.83 ± 0.05 logMAR in the POAG group and 0.91 ± 0.05 logMAR in the PEXG group, and the inter-group difference was not of statistical significance (*p* = 0.29). Both cohorts experienced improvements afterwards, but more moderately in the PEXG group; at 3 months, BCVA reached 0.28 ± 0.04 logMAR and 0.49 ± 0.06 logMAR, respectively, with a significant difference between the two cohorts (*p* = 0.02). The BCVA worsened again in both groups later and diverged significantly at 5 years (*p* = 0.04), with the mean values reaching 0.34 ± 0.08 logMAR and 0.60 ± 0.09 logMAR in the POAG and PEXG groups.

**Figure 4:**
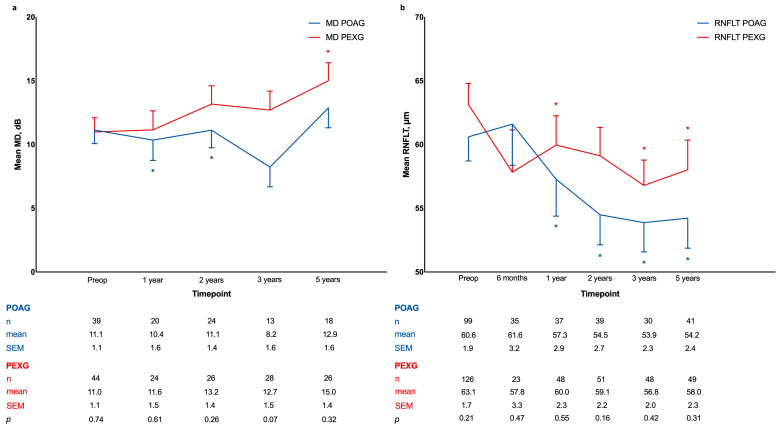
Development of visual field mean defects (**a**) and peripapillary retinal nerve fiber layer thickness (**b**) during 5 years of follow-up after deep sclerectomy in primary open-angle glaucoma and pseudoexfoliation glaucoma groups. MD, mean defect; RNFLT, retinal nerve fiber layer thickness; POAG, primary open-angle glaucoma; PEXG, pseudoexfoliation glaucoma; Preop, preoperative; SEM, standard mean error. Comparison between baseline and follow-ups within groups was performed using Wilcoxon signed-rank test (* indicates *p* < 0.05). Comparison between groups was performed using Mann–Whitney U test; *p* values are reported in last row.

The development of visual field mean defects is shown in Figure 4. At 5 years, MDs remained stable compared to the preoperative values at baseline in the POAG group (12.9 ± 1.6 dB, *p* = 0.08), while values significantly worsened in the PEXG group (15.0 ± 1.4 dB, *p* = 0.02). A statistically significant difference between cohorts was absent at all the analyzed post-surgical timepoints.

### 3.7. Peripapillary Retinal Nerve Fiber Layer Thickness

Mean peripapillary RNFL thickness was 60.6 ± 1.9 µm in the POAG and 63.1 ± 1.7 µm in the PEXG cohort at baseline (Figure 4). At 1 year after surgery, the mean peripapillary RNFL thickness decreased significantly in both POAG (*p* = 0.02) and PEXG (*p* = 0.03) groups compared to baseline, reaching 57.3 ± 2.9 µm and 60.0 ± 2.3 µm, respectively. Afterwards, it remained stable in both cohorts, with no statistically significant difference found using the Wilcoxon signed-rank test between measured values at 1 and 2 years (POAG: *p* = 0.46, PEXG: *p* = 0.68), at 2 and 3 years (POAG: *p* = 0.40, PEXG: *p* = 0.14), and at 3 and 5 years postoperatively (POAG: *p* = 0.98, PEXG: *p* = 0.13). Overall, after 5 years, the mean peripapillary RNFL thickness had a reduction of 11% ± 5% in the POAG cohort (*p* < 0.001) and 8% ± 5% in the PEXG cohort (*p* < 0.001) compared to preoperative values. Nevertheless, a statistically significant difference between groups was absent at every timepoint. Furthermore, a correlation analysis indicated no significant association between changes in IOP and changes in peripapillary RNFL thickness after 5 years (Pearson correlation coefficient: 0.23, *p* = 0.05).

Because of the statistically significant difference in mean age at surgery between the POAG and PEXG groups (*p* < 0.001), a correlation analysis was conducted to understand if this factor affected any difference in outcomes. The results of this analysis are shown in Table 4. The Pearson correlation coefficient was calculated for the age at surgery and each of the following differences in outcomes after 5 years: the mean IOP, the mean number of IOP-lowering medications, the total number of postoperative interventions, the total number of secondary major glaucoma surgeries, the mean BCVA, MDs, and the mean peripapillary RNFL thickness. The point biserial correlation coefficient was calculated between the age at surgery and the achievement of qualified and complete surgical success at 5 years. Only the difference in the MD after 5 years was found to be weakly [26] positively associated with patient age (0.49, *p* = 0.02). No other factor showed any significant correlation.

## 4. Discussion

Our analysis demonstrated that in the short term after surgery, DS is equally effective in decreasing IOP and the number of IOP-lowering medications in POAG and PEXG cases. This led to comparable results for complete and qualified success at 1 and 3 years after surgery while stabilizing BCVA and visual field MD in both groups. However, in the medium term, the percentage of PEXG cases meeting success criteria decreased strongly and the difference to the POAG group became statistically significant at 5 years after surgery. Also, the number of eyes needing secondary glaucoma surgery was larger in the PEXG group compared to the POAG group (*p* = 0.01). Additionally, RNFL decrease continued until 1 year after DS in both groups, stabilizing thereafter in both POAG and PEXG eyes.

These results are similar to previous findings by Ollikainen et al. [17], suggesting no significant difference in IOP values between 31 POAG and 37 PEXG eyes 3 years after DS. Another study by Studeny et al. [18] found a significant IOP reduction in 10 PEXG patients 2 years after DS performed with a T-flux implant, with mean values decreasing from 36.8 ± 8.7 mmHg to 14.8 ± 2.4 mmHg (median values, ±standard deviation (SD), *p* < 0.001). The change in IOP was larger than our observed reduction at the same timepoint, which accounted for a decrease from 24.3 ± 0.8 mmHg to 15.2 ± 1.0 mmHg (*p* < 0.001). In both cohorts, the postoperative phase of scarring activity induced a delayed increase in IOP after an initial period of rapid IOP reduction [27]. The strong IOP reduction in the early postoperative period may be due to the surgical strategy used in our clinic. During surgery, strong filtration is allowed from the beginning, aiming at borderline hypotensive IOP levels at the end of the procedure. Usually, only a few scleral flap sutures are used with very low tension to minimize interference with aqueous humor flow. Excessive hypotension is not common and manageable if present. Nevertheless, laser suture lysis and 5-FU injections are a common part of the postoperative regimen in our clinic.

Our results concerning the necessity for the application of IOP-lowering medications after surgery were similar to a previous study by Ollikainen et al. [19], which also found no difference regarding the reduction in the number of glaucoma medications between 31 POAG and 37 PEXG eyes up to 1 year after DS. The change in the PEXG group at 5 years after DS (medication reduction from 3.4 ± 0.1 to 1.7 ± 0.2, *p* < 0.001) was more moderate compared to a study by Mendrinos et al. [20], where 22 PEXG eyes had a reduction from 2.4 ± 0.67 to 0.59 ± 0.85 after a follow-up of 48.5 ± 12.2 months (mean values, ±SD, *p* < 0.0001).

The percentage of eyes undergoing postoperative interventions was comparable in both groups. This was similar to a previous study by Drolsum [21] that reported a non-significant difference in cases which underwent goniopuncture in 27 POAG (30%; mean follow-up of 43 months) and 28 PEXG eyes (32%; mean follow-up of 45 months). In comparison, our observed percentage of cases with goniopunctures was 28% and 37% in the POAG and PEXG cohorts, respectively, which also lacked a difference in statistical significance between both groups (chi-square test: *p* = 0.10).

However, our observations regarding differences in re-operation rates showed that 8% of POAG eyes and 21% of PEXG eyes (*p* = 0.01) underwent secondary major glaucoma surgery during the 5 years of follow-up after DS, which suggests an overall worse postoperative course in the PEXG cohort. In comparison, Rekonen et al. [22] reported a 10% rate in POAG eyes and 18% rate in PEXG eyes during a follow-up of 18 months after DS (no statistically significant difference between groups). Interestingly, at 18 months after DS, the different rate of necessary major secondary glaucoma surgery between our POAG and PEXG cohorts was also not of statistical significance (4% POAG; 6% PEXG; chi-square test: *p* = 0.42). Only analysis of longer follow-up results in our study showed diverging re-operation rates between POAG and PEXG cohorts with differences in statistical significance. Drolsum [21] and Mendrinos et al. [20], who analyzed longer postoperative follow-up periods, did not report detailed information concerning re-operation rates.

The analysis regarding the progression of visual field defects was affected by the statistically significant older mean age at surgery of the PEXG cohort. Studies by Konstas et al. [28] and Nakano et al. [29] have reported that PEXG patients tend to be older than POAG patients, indicating that this age difference may be an epidemiological difference rather than an inclusion bias. Age did not influence other outcomes. Although the correlation between this factor and the changes in SAP results after 5 years was weak, it may have nonetheless played a role in shaping the observed findings, contributing to the assessed worsened visual field defects among PEXG patients 5 years after DS compared to preoperative values. Nevertheless, after 5 years, no statistically significant difference was found between groups for this outcome, which is relevant in the overall analysis, as a less compromised visual field is directly correlated with a higher quality of life [30]. A comparable reduction in peripapillary RNFL thickness was recorded for both cohorts, which stabilized approximately after the first postoperative year and was not associated with the resulting IOP changes after surgery. The absence of this correlation has already been described in the literature [31]. In a prospective trial by Rebolleda et al. [32], visual field global indices and peripapillary RNFL thickness were found to be comparable to preoperative values 6 months after DS in 34 patients with unspecified glaucoma. Our initial observations for these outcomes in PEXG eyes were similar, with no significant postoperative changes at 6 months in peripapillary RNFL thickness (*p* = 0.19) but with later worsening courses for peripapillary RNFL thickness (*p* < 0.001) and for the mean defect values of SAP (*p* = 0.02) after 5 years.

Similarly, the performed analysis showed worse outcomes for success levels among PEXG patients 5 years after DS, as POAG patients tended to achieve higher rates for both qualified and complete success. These outcomes may underestimate existing results due to the large rate of dropouts during follow-up. This assumes that patients with more favorable outcomes may be less likely to attend control examinations, as has already been described for other ophthalmic procedures before, such as strabismus surgery [33]. It also assumes that patients who experience worse subjective symptoms may indeed have less favorable objective outcomes, as Viswanathan et al. have observed for visual field deterioration in particular [34], and might therefore worsen observed results if they are more likely to attend follow-up. The aforementioned study by Studeny et al. [18] indicated complete and qualified success rates of 85% and 100%, respectively, in 20 PEXG eyes 1 year after DS, which were noticeably higher than ours at the same timepoint (39% for complete success and 55% for qualified success). Furthermore, Ollikainen et al. [17] reported no significant difference for both complete and qualified success rates between 31 POAG eyes (74% and 74%, respectively) and 37 PEXG eyes (73% and 73%) 3 years after surgery. These values are higher than our findings at the same timepoint but are in line with our results concerning a comparison between both groups, as we also could not find differences in statistical significance between groups 3 years after DS. A longer trial by Drolsum [21] showed the absence of statistically significant differences concerning the achievement of complete success after a mean follow-up time of approximately 4 years, with 50.0% in PEXG eyes compared to 33.3% in POAG eyes respecting the criteria.

The inferior success rates, together with worse BCVA, ultimately led to overall worse results of DS in the PEXG cohort compared to the POAG cohort at 5 years after surgery, even though other clinical outcomes were indeed similar between both groups. Other studies indicated before that DS may be safe and useful for PEXG eyes in the medium term [17,18,19,20,21,22]. The herein presented results lightly hint towards DS being more beneficial in POAG eyes compared to PEXG over a longer follow-up period. Other surgical options for PEXG patients include TE. However, there are also conflicting results regarding the efficacy of TE in PEXG eyes [35,36,37], suggesting that PEXG may be more difficult to treat than POAG, regardless of therapeutic approach.

Our herein presented analysis provides detailed information about the development of anatomical (peripapillary RNFL thickness) and functional parameters (SAP indices) after DS, as well as a large quantity of other clinical data that are frequently reported in similar but mostly smaller cohorts. Moreover, we followed patients over a longer period of time, which is essential given the chronic nature of glaucoma. We also analyzed a sizeable number of patients with data originating from only one eye per participant included in the analysis, which was useful to provide statistically significant and higher quality information. Weaknesses of the study include the lack of analysis of postoperative complications, the retrospective, non-randomized, unblinded, and unicentric design, the number of patients lost to follow-up, and the multiple number of surgeons performing surgery. DS is a procedure that has been frequently performed by many surgeons at the Bern University Hospital over the years. This may lead to variations in the procedure performed. However, we did not find a high variability of postoperative outcomes in terms of IOP, medications, and success rate. Performing the herein described analyses in a larger cohort coming from multiple centers in a prospective manner could be a goal of future studies.

## 5. Conclusions

DS has been proven to provide comparable outcomes in PEXG and POAG eyes regarding IOP, the number of IOP-lowering medications, visual field defects, and peripapillary RNFL thickness 5 years after surgery. However, PEXG eyes were significantly more likely to require subsequent major surgical glaucoma interventions, and the complete and qualified success rates were lower after 5 years of follow-up compared to POAG eyes. Based on the results of our study, it can be argued that the long-term success of DS in eyes suffering from PEXG is lower than in those suffering from POAG. In our case, this will influence our strategy when planning surgical interventions for the treatment of glaucoma (POAG and PEXG) in the future. The presented results may help surgeons and patients make more informed choices about their treatment options. Nevertheless, more studies with larger patient cohorts are needed to evaluate this procedure and other treatment options to provide better targeted surgical care for PEXG patients.

## Figures and Tables

**Table 1:** Baseline demographic and clinical characteristics of the study participants.

	POAG	PEXG	*p*
	(*n* = 109)	(*n* = 153)	
Age, yrs	68.8 ± 1.0	74.2 ± 0.6	**<0.001 ^a^**
Male sex, %	56%	36%	**0.001 ^b^**
Right eye, %	44%	52%	0.23 ^b^
Eyes with history of previous glaucoma surgery, %	2.8%	2.0%	0.67 ^b^
Mean IOP, mmHg	22.8 ± 0.7	24.3 ± 0.8	0.10 ^a^
Mean number of IOP-lowering medications, *n*	3.1 ± 0.1	3.4 ± 0.1	0.11 ^a^
Mean BCVA, LogMAR	0.25 ± 0.02	0.37 ± 0.04	0.06 ^a^
Mean MD, dB	11.1 ± 1.1	11.0 ± 1.1	0.74 ^a^
Mean peripapillary RNFL thickness, µm	60.6 ± 1.9	63.1 ± 1.7	0.21 ^a^

POAG, primary open-angle glaucoma; PEXG, pseudoexfoliation glaucoma; IOP, intraocular pressure; BCVA, best corrected visual acuity; MD, mean defect; RNFL, retinal nerve fiber layer. *p* values less than 0.05 are in bold. ^a^ Mann–Whitney U test; ^b^ chi-square test.

**Table 2:** Development of mean intraocular pressure and number of glaucoma medications during 5 years of follow-up after deep sclerectomy, including patients who underwent secondary glaucoma surgeries.

Timepoint	IOP, mmHg			Number of Medications, *n*		
	POAG	PEXG	*p* ^a^	POAG	PEXG	*p* ^a^
Preoperative	22.8 ± 0.7	24.3 ± 0.8	0.10	3.1 ± 0.1	3.4 ± 0.1	0.11
1 day	4.9 ± 0.4	6.1 ± 0.6	0.46	0.1 ± 0.04	0.1 ± 0.05	0.91
1 month	10.5 ± 0.5	11.4 ± 0.5	0.12	0.02 ± 0.02	0.04 ± 0.02	0.31
3 months	13.3 ± 0.8	13.6 ± 0.7	0.54	0.1 ± 0.1	0.1 ± 0.1	0.75
6 months	13.3 ± 0.6	13.2 ± 0.8	0.96	0.3 ± 0.1	0.5 ± 0.1	0.14
1 year	13.9 ± 1.0	16.2 ± 1.0	0.05	0.7 ± 0.2	0.8 ± 0.2	0.41
2 years	13.7 ± 0.6	15.2 ± 1.0	0.54	1.0 ± 0.2	1.1 ± 0.2	0.58
3 years	13.6 ± 0.7	16.8 ± 1.4	0.19	1.6 ± 0.3	1.4 ± 0.2	0.68
5 years	13.3 ± 0.6	16.6 ± 1.2	0.17	1.7 ± 0.3	1.7 ± 0.2	0.99

IOP, intraocular pressure; POAG, primary open-angle glaucoma; PEXG, pseudoexfoliation glaucoma. ^a^ Comparison between two groups was performed using Mann–Whitney U test.

**Table 3:** Number of postoperative interventions during 5 years of follow-up after deep sclerectomy.

Postoperative Intervention	POAG		PEXG		*p*
	Number of Eyes	%	Number of Eyes	%	
Iridoplasties	4	4	16	7	0.55
Bleb needlings	11	9	24	11	0.75
Goniopunctures	34	28	63	37	0.24
5-FU injections	78	32	115	35	0.97
Open surgical revisions	6	6	15	10	0.21
Total	133	51	233	61	0.35

POAG, primary open-angle glaucoma; PEXG, pseudoexfoliation glaucoma; 5-FU, 5-fluorouracil. Comparison between two groups were performed using chi-square test.

**Table 4:** Analysis of correlation between age at surgery and 5-year outcomes.

Outcome	Correlation Coefficient	*p* *
Δ mean IOP, mmHg	0.009 ^a^	0.93
Δ mean number of IOP-lowering medications, *n*	−0.12 ^a^	0.26
Δ mean BCVA, LogMAR	0.14 ^a^	0.19
Δ mean MD, dB	0.49 ^a^	**0.02**
Δ mean RNFL thickness, µm	−0.07 ^a^	0.52
Total number of postoperative interventions, *n*	−0.06 ^a^	0.38
Total number of secondary glaucoma surgeries, *n*	−0.05 ^a^	0.42
Number of eyes that achieved complete success	0.009 ^b^	0.93
Number of eyes that achieved qualified success	0.13 ^b^	0.22

IOP, intraocular pressure; BCVA, best corrected visual acuity; MD, mean defect; RNFL, retinal nerve fiber layer. *p* values of less than 0.05 are in bold. ^a^ Pearson; ^b^ point biserial; * two-tailed test.

## Data Availability

The datasets used for this study are available from the corresponding author upon reasonable request.

## References

[1] Höhn R., Pfeiffer N. (2017). Klassifikation, Genetik und Epidemiologie der Glaukome. Klin. Monbl. Augenheilkd..

[2] Weinreb R.N., Aung T., Medeiros F.A. (2014). The pathophysiology and treatment of glaucoma: A review. JAMA.

[3] Gedde S.J., Lind J.T., Wright M.M. (2021). American Academy of Ophthalmology Preferred Practice Pattern glaucoma panel. Primary open-angle glaucoma Preferred Practice Pattern^®^. Ophthalmology.

[4] Tham Y.C., Li X., Wong T.Y., Quigley H.A., Aung T., Cheng C.Y. (2014). Global prevalence of glaucoma and projections of glaucoma burden through 2040: A systematic review and meta-analysis. Ophthalmology.

[5] Elhawy E., Kamthan G., Dong C.Q., Danias J. (2012). Pseudoexfoliation syndrome, a systemic disorder with ocular manifestations. Hum. Genom..

[6] Hicks P.M., Siedlecki A., Haaland B., Owen L.A., Au E., Feehan M., Murtaugh M.A., Sieminski S., Reynolds A., Lillvis J. (2021). A global genetic epidemiological review of pseudoexfoliation syndrome. Explor. Med..

[7] Ritch R., Schlötzer-Schrehardt U., Konstas A.G. (2003). Why is glaucoma associated with exfoliation syndrome?. Prog. Retin. Eye Res..

[8] Lee D.A., Higginbotham E.J. (2005). Glaucoma and its treatment: A review. Am. J. Health-Syst. Pharm..

[9] Rao A., Cruz R.D. (2022). Trabeculectomy: Does it have a future?. Cureus.

[10] Razeghinejad M.R., Fudemberg S.J., Spaeth G.L. (2012). The changing conceptual basis of trabeculectomy: A review of past and current surgical techniques. Surv. Ophthalmol..

[11] Rulli E., Biagioli E., Riva I., Gambirasio G., De Simone I., Floriani I., Quaranta L. (2013). Efficacy and safety of trabeculectomy vs nonpenetrating surgical procedures: A systematic review and meta-analysis. JAMA Ophthalmol..

[12] Richardson-May J., Alnuaimi R., Elbably A., Walker L., Thulasidharan S., Dacombe R., Jacob A. (2023). Our experience of deep sclerectomy at a tertiary center in the United Kingdom over 14 years. Cureus.

[13] Klemm M. (2015). Tiefe Sklerektomie. Eine Alternative zur Trabekulektomie. Ophthalmologe.

[14] Heijl A., Bengtsson B., Hyman L., Leske M.C., Early Manifest Glaucoma Trial Group (2009). Natural history of open-angle glaucoma. Ophthalmology.

[15] Gottanka J., Flügel-Koch C., Martus P., Johnson D.H., Lütjen-Drecoll E. (1997). Correlation of pseudoexfoliative material and optic nerve damage in pseudoexfoliation syndrome. Investig. Ophthalmol. Vis. Sci..

[16] Suominen S.M., Harju M.P., Vesti E.T. (2016). Deep sclerectomy in primary open-angle glaucoma and exfoliative glaucoma. Eur. J. Ophthalmol..

[17] Ollikainen M.L., Puustjärvi T.J., Rekonen P.K., Uusitalo H.M., Teräsvirta M.E. (2011). Mitomycin C-augmented deep sclerectomy in primary open-angle glaucoma and exfoliation glaucoma: A three-year prospective study. Acta Ophthalmol..

[18] Studeny P., Baxant A.D., Vranova J., Kuchynka P., Pokorna J. (2017). Deep sclerectomy with nonabsorbable implant (T-Flux) in patients with pseudoexfoliation glaucoma. J. Ophthalmol..

[19] Ollikainen M., Puustjärvi T., Rekonen P., Uusitalo H., Teräsvirta M. (2010). Mitomycin-C-augmented deep sclerectomy in patients with primary open-angle glaucoma and exfoliation glaucoma: A 1-year prospective study. Acta Ophthalmol..

[20] Mendrinos E., Mansouri K., Mermoud A., Shaarawy T. (2009). Long-term results of deep sclerectomy with collagen implant in exfoliative glaucoma. J. Glaucoma.

[21] Drolsum L. (2006). Longterm follow-up after deep sclerectomy in patients with pseudoexfoliative glaucoma. Acta Ophthalmol. Scand..

[22] Rekonen P., Kannisto T., Puustjärvi T., Teräsvirta M., Uusitalo H. (2006). Deep sclerectomy for the treatment of exfoliation and primary open-angle glaucoma. Acta Ophthalmol. Scand..

[23] Hoffer K.J., Aramberri J., Haigis W., Olsen T., Savini G., Shammas H.J., Bentow S. (2015). Protocols for studies of intraocular lens formula accuracy. Am. J. Ophthalmol..

[24] Anand N., Kumar A., Gupta A. (2011). Primary phakic deep sclerectomy augmented with mitomycin C: Long-term outcomes. J. Glaucoma.

[25] Mermoud A., Schnyder C.C. (2000). Nonpenetrating filtering surgery in glaucoma. Curr. Opin. Ophthalmol..

[26] Miot H.A. (2018). Correlation analysis in clinical and experimental studies. J. Vasc. Bras..

[27] Delarive T., Rossier A., Rossier S., Ravinet E., Shaarawy T., Mermoud A. (2003). Aqueous dynamic and histological findings after deep sclerectomy with collagen implant in an animal model. Br. J. Ophthalmol..

[28] Konstas A.G., Tsatsos I., Kardasopoulos A., Bufidis T., Maskaleris G. (1998). Preoperative features of patients with exfoliation glaucoma and primary open-angle glaucoma. The AHEPA study. Acta Ophthalmol. Scand..

[29] Nakano H., Togano T., Sakaue Y., Suetaka A., Iikawa R., Nakano R., Fukuchi T. (2020). Clinical features of patients with exfoliation glaucoma requiring surgical intervention. J. Ophthalmol..

[30] Rossi G.C.M., Milano G., De Silvestri A., Savini L., Bosi C., Gambini G., Rama P. (2023). Correlation between visual field index and quality of life in glaucoma patients: A new tool to screen quality of life perception?. Front. Med..

[31] Koenig S.F., Hirneiss C.W. (2021). Changes of neuroretinal rim and retinal nerve fiber layer thickness assessed by optical coherence tomography after filtration surgery in glaucomatous eyes. Clin. Ophthalmol..

[32] Rebolleda G., Muñoz-Negrete F.J., Noval S. (2007). Evaluation of changes in peripapillary nerve fiber layer thickness after deep sclerectomy with optical coherence tomography. Ophthalmology.

[33] Daly C.M., Dembinski R.L., Kraus C.L. (2021). Factors leading to loss to follow-up after strabismus surgery in children. J. AAPOS.

[34] Viswanathan A.C., McNaught A.I., Poinoosawmy D., Fontana L., Crabb D.P., Fitzke F.W., Hitchings R.A. (1999). Severity and stability of glaucoma: Patient perception compared with objective measurement. Arch. Ophthalmol..

[35] Jerndal T., Kriisa V. (1974). Results of trabeculectomy for pseudo-exfoliative glaucoma. A study of 52 cases. Br. J. Ophthalmol..

[36] Li F., Tang G., Zhang H., Yan X., Ma L., Geng Y. (2020). The effects of trabeculectomy on pseudoexfoliation glaucoma and primary open-angle glaucoma. J. Ophthalmol..

[37] Popovic V., Sjöstrand J. (1999). Course of exfoliation and simplex glaucoma after primary trabeculectomy. Br. J. Ophthalmol..

